# The Accuracy of Custom-Made Milled Metal Posts as Compared to Conventional Cast Metal Posts

**DOI:** 10.3390/dj12100309

**Published:** 2024-09-28

**Authors:** Tabarak M. AL-Rubaye, Emad S. Elsubeihi

**Affiliations:** 1 College of Dentistry, Ajman University, Ajman P.O. Box 346, United Arab Emirates; 202011374@ajmanuni.com; 2 Center of Medical and Bio-Allied Health Sciences Research, Ajman University, Ajman P.O. Box 346, United Arab Emirates

**Keywords:** post, custom-made, milled post, half-digital, cast post, accuracy

## Abstract

Aim: The aim of this study was to compare the fitting accuracy of custom-made metal posts and cores fabricated by half-digital and milling technique to that of conventional cast posts fabricated by direct technique. Methods: Sixteen extracted single-rooted teeth were endodontically treated followed by post space preparation. A direct resin post and core pattern was made for each tooth and used for the fabrication of two posts (n = 16). Each post resin pattern was digitized with a laboratory scanner and used for the fabrication of a milled cobalt–chrome (Co–Cr) alloy post, while the direct resin pattern, after scanning, was cast in a Co–Cr alloy to produce a cast post. Each post was seated on its respective tooth and evaluated using microcomputed tomography. The following variables were evaluated: total space volume between the post and root canal, the volume and distance of the apical gap between each post and the remaining apical root canal filling, as well as the distance and surface area of the space between the post and lateral root canal wall at four determined points along the length of each post. Results: The results revealed that half-digital and milled posts had a statistically significantly higher total space volume (*p* < 0.05), apical gap volume (*p* < 0.02) and distance (*p* < 0.02), as well as a higher surface area of space between the post and root canal wall at the cervical area as compared to the cast post (*p* < 0.05). Conclusions: This study demonstrated that the fitting accuracy of cast posts was more accurate than posts fabricated with half-digital and milling technique.

## 1. Introduction

Post and core restorations have been used over the years to restore missing coronal tooth structure, thus providing retention and support for extra-coronal restorations used to restore endodontically treated teeth. Generally, post and core restorations can be classified into prefabricated and custom-made posts. While there is increased interest in using prefabricated posts over custom-made posts for the restoration of endodontically treated teeth [1], studies have indicated that clinicians still use both types of posts [2]. The choice of post depends on several factors that include tooth-related factors as well as the knowledge, experience, and training of the clinician [3]. Studies have reported that prosthodontists favor the use of custom-made cast metal posts, particularly in teeth with an extensive loss of coronal tooth structure [4,5]. In addition, custom-made posts provide a close adaptation between the post and the prepared post space in flared roots, as well as in roots with oval, elliptical or c-shaped canals [6,7]. The adaptation of the post to the prepared root canal has been regarded as an essential requirement to achieve a homogeneous, uniform, thin cement layer in order to optimize retention [8,9,10,11,12] and prevent the development of unfavorable forces that may lead to failure [13,14,15,16].

Traditionally, custom-made posts are fabricated using the lost wax technique. In this technique, posts are cast in metal alloy following the fabrication of a post pattern made using either indirect or direct technique [17,18]. In the indirect technique, an impression of the prepared post space is made using elastomeric impression material, followed by the fabrication of a stone model upon which a wax or resin post pattern is fabricated in the laboratory [17]. On the other hand, in the direct technique the clinician fabricates the post pattern directly on the prepared tooth in the patient’s mouth [18]. Historically, custom-made posts were cast using noble alloys. However, because of the increased prices of noble alloys, custom-made posts are currently made of non-noble alloys including base metal cobalt–chrome (Co–Cr), nickel–chrome (Ni–Cr), and titanium alloys [18,19,20]. In vitro studies [17] found no difference in the fitting accuracy of cast base metal custom-made posts fabricated using the direct or indirect techniques. Furthermore, a randomized clinical trial found that both techniques produced clinically acceptable posts made of base metal alloy, although the indirect technique produced shorter posts at the apical region when compared to those produced by the direct technique [18].

Advances in digital dentistry have increased the popularity of these techniques in the fabrication of dental restorations, including post and core restorations [21]. Currently, there are two main digital approaches used for the fabrication of custom-made posts. These are half-digital (also known as semi-digital, partial-digital, or indirect technique) and fully digital (also known as direct) technique [20,22,23,24,25,26,27]. In the half-digital technique, a conventional impression of the prepared post space or a directly made wax or resin pattern of the post space is scanned, and the restoration is then made using a subtractive milling or additive 3D printing technique [20,25]. In the full-digital technique, on the other hand, the post space is prepared with a special drill and made to conform to the shape of a specific scan post, which is then used to scan the post space preparation directly in the patient’s mouth [21,28]. The post and core restoration is then either milled or 3D-printed with the chosen material [29]. With the improved depth of scan of recent intra-oral scanners, it has been suggested that the post space preparation can be directly scanned, without a scan post, allowing the preparation of the post space based on the shape of the root canal and the remaining tooth structure [30]. However, recent studies have shown that the direct scanning of the post space preparation without the scan post produced inaccurate custom-made posts as compared to the full digital approach using scan posts [30,31].

Different materials have been suggested to fabricate custom-made post and cores using the digital approach, including glass fiber-reinforced composites [24,32,33], zirconia [22,23,31,34,35], the hybrid ceramic Vita Enamic and the nano-ceramic resin composite Lava Ultimate [27], polyetheretherketone (PEEK) [36], titanium [19,37], and base metal Co–Cr alloys [9,38].

Few studies have evaluated the accuracy of digitally fabricated custom-made posts and cores made of base metal alloys. One study, using microcomputed tomography, found that the accuracy of cast custom-made posts made of base metal alloy using conventional lost-wax technique was more accurate than that using selective laser melting (SLM) based 3D printing technique [38]. Another study [9] evaluated the apical gap and retention of custom-made base metal posts fabricated using conventional, half-digital, and full-digital techniques. In that study [9], cast posts were made using direct acrylic resin patterns, whereas posts made using the digital workflow were milled from Co–Cr base metal alloy. It was found that cast posts produced using the conventional technique provided a smaller apical gap, as measured by parallel digital radiographs, and a higher retention when compared to posts fabricated using a digital workflow [9].

To our knowledge, no study evaluated the accuracy of fit of milled custom-made posts of Co–Cr base metal alloy fabricated using half-digital and milled technique. Thus the aim of this study was to evaluate the fitting accuracy of milled custom-made Co–Cr base metal alloy posts fabricated using half-digital technique as compared to cast Co–Cr base metal alloy posts fabricated using the conventional lost wax technique. The null hypothesis was that, when evaluated using µCT, there are no differences in total space volume, apical gap volume, apical distance, and space between the lateral side of the post and root canal in custom-made metal posts fabricated using half-digital and milled technique as compared to cast custom-made metal posts.

## 2. Materials and Methods

### 2.1. Study Design

**Figure 1:**
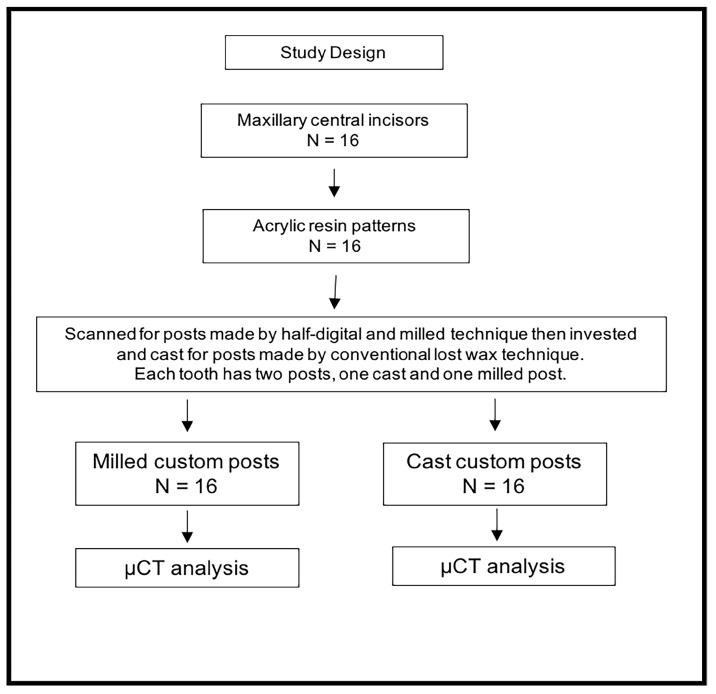
Study design showing groups and number of posts in each group. N = sample size. µCT = microcomputed tomography.

A total of 16 extracted single-root maxillary central incisors were obtained, and two custom posts were made for each tooth (*n* = 16). The first post was a cast custom post fabricated using conventional lost wax technique, while the second post was a milled custom post fabricated using half-digital technique. Each post was seated in its respective tooth and scanned using µCT to compare the fitting accuracy of the custom cast and custom milled posts according to the study design shown in Figure 1.

### 2.2. Selection and Preparation of Samples

Teeth were cleaned by hand scaling of any deposits and calculus. Afterwards, the teeth were inspected with 3.0× magnification loupes (UNIVET, Rezzato (BS), Italy) for any cracks and fractures. Periapical radiographs were taken for each tooth using digital X-ray in the facio-lingual and mesio-distal directions to ensure a similar single root canal morphology. Inclusion criteria included single-root sound maxillary central incisors with a root length of at least 14 mm. The selected teeth have no root canal treatment and are free of any signs of internal or external resorption. We selected teeth that, as much as possible, had a similar morphology and dimensions, as determined by measurements using a digital caliper (Insize digital caliper, Standard Model, Santana Sao Paulo, Brazil) as well as radiographic examination.

#### 2.2.1. Root Canal Treatment

Endodontic access preparation was performed using standard methods (Cavity access Z set, Dentsply Sirona, Charlotte, NC 28277, USA). The patency of the apical foramen was confirmed using a size #10 K-type file (SybronEndo^®^, GAM Mexico City, CP 07580, Mexico). The working length was determined to be 1.0 mm shorter than where the initial file exited the apical foramen. This was done to ensure that the apical preparation was completed within 1.0 mm of the apical foramen. Biomechanical instrumentation was performed using a wave-one gold rotary size large file 45/05 (Dentsply Sirona, AG Baden, Switzerland). As part of shaping and cleaning, sodium hypochlorite 5% solution (4.0 mL) was used as irrigant along with Endo Activator size large 35/04 (Dentsply Sirona, Charlotte, NC 28277, USA) to facilitate irrigant penetration and mechanical cleansing. A total of 2.0 mL of 17% EDTA (PPH CERKAMED, Stalowa Wola, Poland) was added as a one-minute soak after instrumentation, followed by a silane rinse. Drying of root canals was accomplished with paper points (Dentsply Sirona, Charlotte, NC 28277, USA). All teeth were fitted with gutta-percha cones large size 45/05 (Dentsply Sirona, AG Baden, Switzerland). A single cone obturation technique was used to obturate the root canals with gutta-percha and sealer (AH Plus sealer, Dentsply DeTrey GmbH, Konstnaz, Germany).

#### 2.2.2. Post Space Preparation

Ten millimeters of gutta-percha was removed mechanically using Gates Glidden drills size 2 and 3 (AZDENT, Zhengzhou, China) from each canal at least 48 h after obturation. The ParaPost^®^ XP System (Coltène, Whaledent^®^, Boca Raton, FL, USA) drills from size 4 (0.040/1.00 mm) to size 6 (0.060/1.50 mm) were used, respectively, to prepare the final post space for each tooth. Following post space preparation, periapical radiographs were taken for each tooth to verify the post preparation length and the remaining gutta percha. On the coronal end of each tooth, a proximal box of 2.0 mm length, 3.0 mm width, and 2.0 mm depth was made using tapered fissure carbide bur in order to help standardize the position of each post in the respective post space during the seating of final posts. Root canals were then rinsed with saline and dried with paper points.

#### 2.2.3. Post Resin Pattern Fabrication

A plastic post (Uniclip 0.8 mm, Dentsply, Maillefer, Ballaigues, Switzerland) was selected and adjusted to fit loosely in the prepared post space. Petroleum jelly was lightly applied to each post space and wiped with paper points. Auto-polymerizing acrylic resin (Pattern ResinTM LS, GC America, Alsip, IL, USA) was then applied to each plastic post using a micro-brush and inserted into each post space to fabricate a post resin pattern using the direct technique. Once polymerized, the post resin pattern was removed and examined for any deficiencies or voids. A core measuring 4.0 mm in height was then added to each post and included the box made on the coronal end of each tooth.

#### 2.2.4. Scanning of Post Resin Pattern

Each post resin pattern was attached to a sprue former at the coronal end of the core, placed on a flat plate, and sprayed with scanning spray (NHT HIGH TECHNOLOGY, Incheon, Republic of Korea). Post resin patterns were then scanned using a laboratory 3D scanner (Ceramill map 400, AmannGirrbach AG, Maeder, Vorarlberg, Austria) to acquire a digital 3D model. Afterwards, the digital model was converted to a Stereolithography file (STL) for the fabrication of a milled post using a five-axis milling machine (Ceramill Motion 2, AmannGirrbach AG, Maeder, Vorarlberg, Austria).

#### 2.2.5. Casting of Custom Metal Posts

As soon as scanning of each post resin pattern was completed, the sprue former of each resin pattern was attached to a crucible former (Rapid Ringless System, BEGO, Lincoln RI 02865, USA) and invested in phosphate-bonded investment (Bellavest^®^ SH class 2, BEGO, Bremen, Germany). For the investment, each 160 g of powder was mixed with 10.0 mL of investment liquid and 30.0 mL of water. Mixing of the investment material was first performed with a hand-held spatula for 30 s, and the mixing was then completed in a vacuum mixer at 350 rpm for 60 s. The casting ring was then filled with the investment and left to set for 30 min.

Each invested post core resin pattern was then inserted into a burnout oven and preheated for 60 min at 900 °C to burn out the acrylic resin pattern (Programix 50 Burnout Furnace, Ugin Dentaire, Seyssinet-Pariset, France). Afterwards, the post was cast with dental Type 4 Co–Cr alloy (Wirobond^®^ C, BEGO, Bremen, Germany) using an induction casting machine (Fornax^®^ T, BEGO, Bremen, Germany). After casting, the investment was allowed to cool down to room temperature, and each cast post and core was divested and sandblasted with 50 µ alumina oxide particles at 6 MPa pressure using a sandblasting machine (Basic Master Sandblasting Machine, Renfert, Hilzingen, Germany). Each cast post core was then labelled and tried on its respective prepared tooth.

#### 2.2.6. Milling of Custom Metal Posts

Each milled metal post and core was made using Co–Cr alloy (Ceramill Sintron R71 CoCrMo disc, AmannGribbach AG, Vorarlberg, Austria) in a five-axis milling machine (Ceramill Motion 2, AmannGirrbach AG, Vorarlberg, Austria). The milling of each post and core was achieved using new Roto motion carbide burs with sizes ranging from 0.3, 0.6 and 1.0 to 2.5 mm (Amann Girbbach AG, Maeder, Vorarlberg, Austria). Following the milling process, each post and core was sintered at 1500 °C for 5 h and 10 min in a sintering furnace (Ceramill Argotherm 2, AmannGribbach AG, Maeder, Vorarlberg, Austria), as per the manufacturer’s instructions.

#### 2.2.7. Seating of Custom Metal Posts and Cores

For each tooth, one cast and one milled post and core were passively seated. To verify the fit, each post was sprayed with marking spray (Arti-Spray, Bausch GmbH & Co. KG, 50769 Köln, Germany) and adjusted. The fit of each post and core was considered acceptable if there was no detectable gap between the core and the proximal box made on the coronal end of each root. A periapical radiograph was taken to verify the seating of each post.

### 2.3. Evaluation of Accuracy of Fit of Cast and Milled Post Cores

#### 2.3.1. Designing and Fabricating a Customized Chamber for Positioning of Samples in µCT

In order to standardize the position of each tooth/post assembly in the µ-CT tube, a customized positioning chamber was made for each tooth to fit inside the µCT tube in a standardized position throughout all the scans. A three-dimensional drawing of the chamber, with a diameter of 11.5 mm and a length of 20 mm, was made using 3D SprintTM for CERAMILL software (3D SprintTM for CERAMILL, version 2.12.0, 3D System^®^, USA). In addition, each chamber had handles measuring 5.0 mm in height, 5.0 mm in width, and 7.5 mm in length.

**Figure 2:**
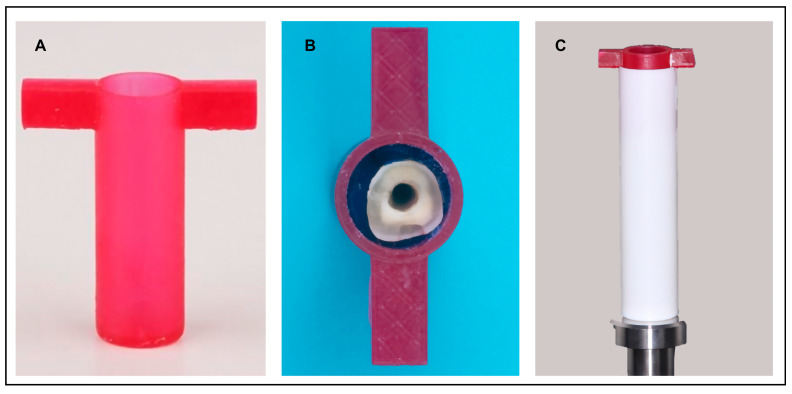
The cured positioning chamber shown in (**A**). Tooth stabilized inside positioning chamber with elastomeric impression material shown in (**B**). Positioning chamber placed inside the scanning tube of the µCT machine shown in (**C**).

The chambers were fabricated using a 3D printing red-colored resin material (NextDent for Ceramill Cast, Amann Girrbach AG, Maeder, Vorarlberg, Austria). For each tooth, one chamber was made. After 3D printing, the chambers were rinsed with 70% isopropyl alcohol in an ultrasonic cleaner for three minutes and soaked in 70% isopropyl alcohol for another 2 min. Each chamber was then dried and placed in a UV light curing unit (LC-3DPrint Box, NextDent 3D Systems, Soesterberg, The Netherlands) for 8 min to complete the polymerization, as per the manufacturer’s instructions (Figure 2A). Each tooth was stabilized inside the positioning chamber (Figure 2B) using elastomeric impression material (Zhermak hydrorise, Badia Polesine (RO), Italy), before placing it in the scanning tube (Figure 2C) of the µCT machine.

#### 2.3.2. Scanning of Samples Using µCT

**Figure 3:**
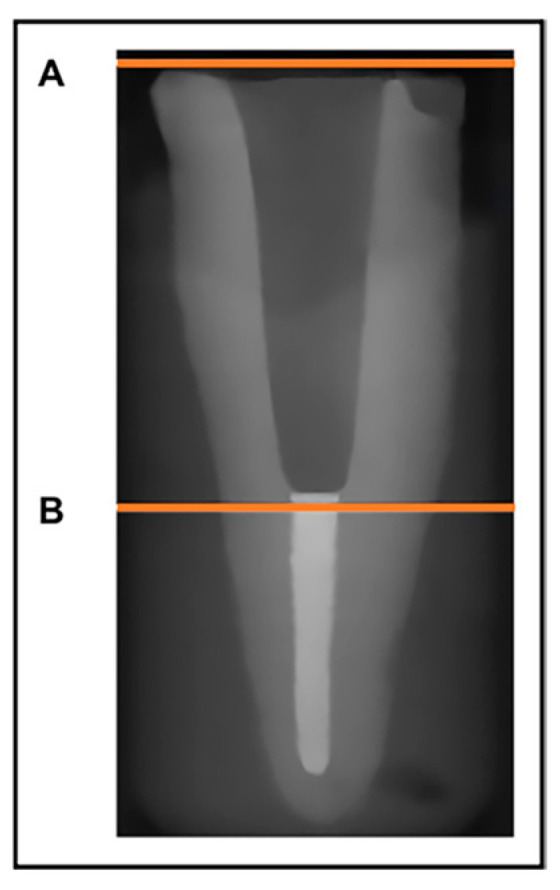
Scout view of scanned sample showing (**A**) the coronal end and (**B**) the apical end of the scanned area.

Samples were scanned with a µCT machine (Scanco Medical μCT100 scanner, Basserdorf, Switzerland). The scanning parameters used for all samples were a native resolution at 90 kV, 44 µA with a voxel size 4.9 µm and a 0.1 mm thick copper filter. Three scans were made for each tooth. These included: (1) tooth with prepared pos space without a post, (2) tooth with cast custom metal post, and (3) tooth with milled custom metal post. Because each tooth was stabilized inside the positioning chamber, it was possible to repeat the three scans of each tooth first without posts and later with cast and then milled posts and cores in a standardized position inside the tube of the µCT machine. The raw images were reconstructed with the software Uct 100 v6.1 (SCANCO Medical micro CT systems, Basserdorf, Switzerland). Subsequently, the area to be scanned was selected in the scout view to start at the most coronal end of the tooth root and end at the coronal end of the remaining gutta perch root canal filling (Figure 3). A grayscale image of the tooth and post was developed in order to visualize and identify the root canal, metal post, gutta-percha, and the gap between the canal walls and the post, as well as that between the post and the apical gutta-percha. As a result, it was possible to compare different measurements at the same µCT slice number of all three scans for each tooth-post assembly. Measurements were performed using a script based on Image Processing Language provided by the µCT manufacturer (SCANCO Medical micro CT systems, Basserdorf, Switzerland).

The following variables were measured: (a) the total volume of the space between the canal and each post, (b) the volume of the apical gap between the end of each post and the remaining gutta-percha, (c) the length of the apical gap between the end of each post and the remaining gutta-percha, (d) the horizontal distance measurement of the space between each post and the canal walls at determined points along the post length, and (e) the surface area of the space between each post and the canal walls at determined points along the post length. All measurements were performed by the same operator and repeated twice, one week apart from each other. The average value of the two measurements for each variable was used for analysis.

#### 2.3.3. Measurement of the Total Volume of the Space between the Root Canal and Each Post

The volume of the post space preparation (VPSP) in mm^3^ was measured on the first scan of the tooth with the post space preparation but without any post and core. Then, the volume of the cast post was measured on the second scan of the tooth with the seated cast post (VCP) in mm^3^. Similarly, the volume of the milled post was measured on the third scan of the tooth with the milled post (VMP) in mm^3^. To calculate the total volume of space between the cast post and post space preparation (Space Volume of Cast post), the volume of the post space preparation (VPSP) was subtracted from the volume of the post space preparation (VPSP) using the formula Space volume of Cast post = VPSP − VCP. Similarly, the total volume of space between the milled post and post space preparation (Space volume of Milled post) was calculated using the following formula: Space volume of Milled post = VPSP − VMP in mm^3^.

The area included during these measurements was standardized so as to extend from the µCT slice of the most coronal tooth structure to the µCT slice of the most coronal remaining gutta-percha. Since each tooth was placed in a positioning chamber during all scans, the areas of measurement of the three scans of each tooth were similar. During analysis, the separation of root dentin from the post was achieved using grayscale thresholds, which had a fixed threshold that was applied to all three scans of each sample. The volume of each measurement (in mm^3^) was obtained by applying the evaluation script based on Image Processing Language provided by the manufacturer.

#### 2.3.4. Measurement of Apical Gap Volume

The apical gap volume (in mm^3^) of cast posts was measured on the second scan in which the cast post was seated in the prepared tooth. Similarly, the apical gap volume of milled posts was measured on the third scan of each sample in which the milled post was seated in the prepared tooth. The measurement of the apical gap volume extended from the µCT slice of the most apical end of each post to the µCT slice of the most coronal end of the remaining gutta-perch root filling. Once the area was determined, the evaluation script was applied, and the apical gap volume was determined for each post.

#### 2.3.5. Length of Apical Gap between Post and Remaining Gutta-Percha Root Filling

The vertical distance between the apical end of each post and the remaining gutta-perch root filling material was measured in mm. Similar to apical gap measurements, the vertical distance of the apical gap was measured from the most apical µCT slice of each post to the most coronal µCT slice of the remaining gutta-percha root filling material. Each measurement was repeated two times on two different occasions that were one week apart, and the average vertical distance of the measurements was calculated and used for analysis.

#### 2.3.6. Measurement of Space between the Posts and Lateral Walls of Root Canal

**Figure 4:**
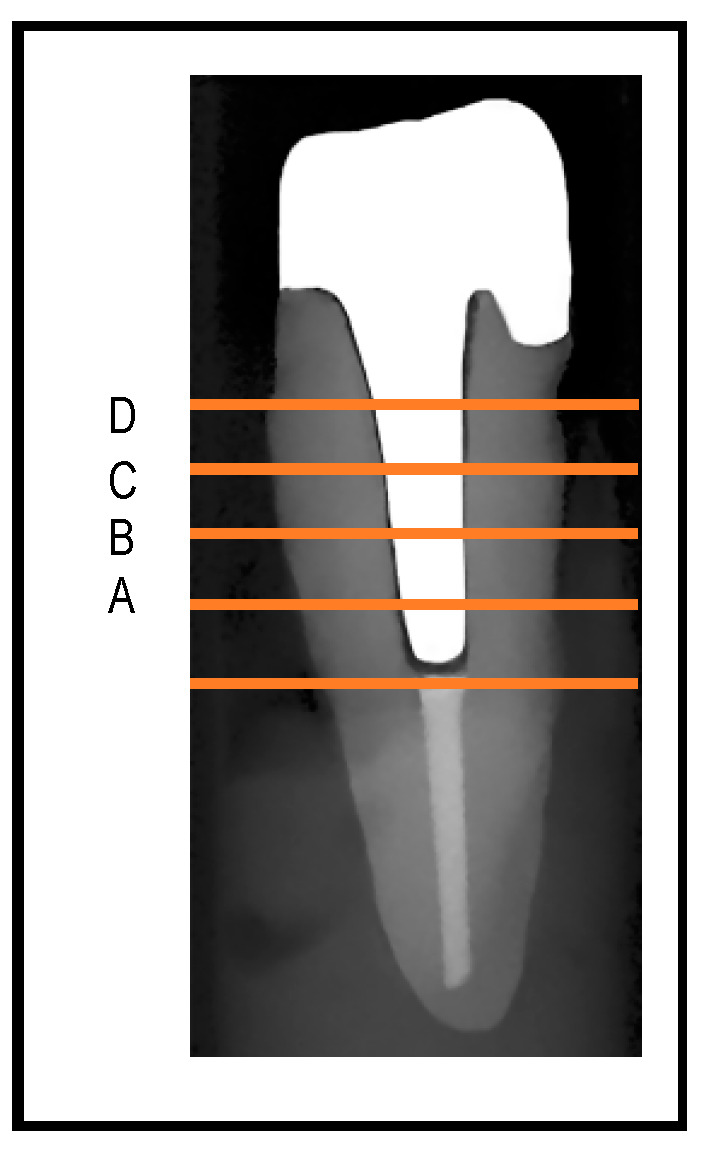
Scanned post sample showing the position of measurements of distances and surface areas along the lateral wall of post. Slice “A” was 2.0 mm coronal to the most coronal end of the remaining gutta-percha root canal filling material. Slices “B”, “C”, and “D” were 1.5 mm apart.

The space between the lateral walls of each post and the prepared root canal was measured on the second scan of each tooth with the seated cast post and the third scan of each tooth with the seated milled posts. These measurements were made at four determined points along the length of the post. The points were determined as follows: point “A” was identified as the µCT slice that was 2 mm coronal to the most coronal end of the remaining gutta-percha root canal filling material. Subsequently, point “B” was 1.5 mm coronal to point “A”, whereas point “C” was 1.5 mm coronal to point “B”, and finally point “D” was 1.5 mm coronal to point “C” (Figure 4).

The standardized position of each point was confirmed in the cast and milled post scans using the slice numbers of the same tooth. Both the distance (in mm) and surface area (in mm^2^) of the space between each post and lateral wall of the prepared canal were performed on cross-sectional µCT slices at the selected four points (A, B, C, and D points) along the length of each post.

On each cross-sectional µCT slice, the software automatically determined the middle of the buccal, mesial, lingual, and distal surfaces of each post. Distance measurements at point A from the lateral wall of each post to the lateral wall of the corresponding root canal were then made at the mid-buccal, mid-mesial, mid-lingual, and mid-distal points determined by the software. Similar distance measurements were made on the other predetermined points along the length of each post, namely B, C, and D.

For surface area measurements, once the canal was contoured on the cross-sectional µCT slice with the post inside the canal, the software automatically provided the surface area of the post and the surface area of the canal in the cross-section. To calculate the surface area of the space between each post and the corresponding canal (in mm^2^) at each predetermined point along the length of the post, the surface area of each post was subtracted from the surface area of the canal on the corresponding µCT slice. Surface area measurements were calculated at the four predetermined areas (A, B, C, and D) along the length of each post. Both distance and surface area measurements were repeated twice, one week apart from each other, and the average of the two measurements was used for analysis.

### 2.4. Statistical Analysis

Data were tested for normality using the Shapiro–Wilk test. The total space volume and apical gap volume (in mm^3^) as well as the apical gap distance (in mm) were analyzed using the Mann–Whitney U test. Distances between posts and canal walls (in mm) and cross-sectional areas between posts and canal walls (in mm^2^) at the four determined points were analyzed using the Kruskal–Wallis test. When differences were detected, Dunn’s post hoc test was used to reveal differences between groups. Intra-class correlation was used to assess the intra-examiner agreement of measurement of the different variables. For all statistical tests, a *p*-value of 0.05 was considered statistically significant. Statistical Package for Social Sciences version 24 (SPSS version 24, 64-bit edition, IBM, Armonk, NY, USA) was used for the analysis.

## 3. Results

### 3.1. Comparison of the Total Space Volume between the Two Groups

**Figure 5:**
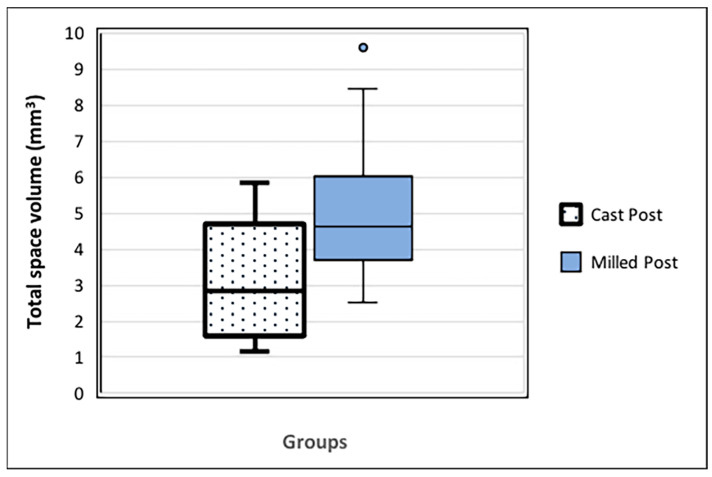
Total space volume (mm^3^) between each post and respective prepared canal. The milled post group demonstrated a higher total space volume value than the cast post group (*p* < 0.05).

The total space volume between the milled post and the prepared root canal (mean 5.06 ± 1.917 mm^3^) was higher than that between the cast post and the prepared root canal (mean 3.071 ± 1.56 mm^3^), as shown in Figure 5. The reliability of the repeated measurements of the total space volume measurements was 0.963 [95% CI 0.925; 0.982]; *p* = 0.0001.

### 3.2. Comparison of Apical Gap Volume between the Two Groups

**Figure 6:**
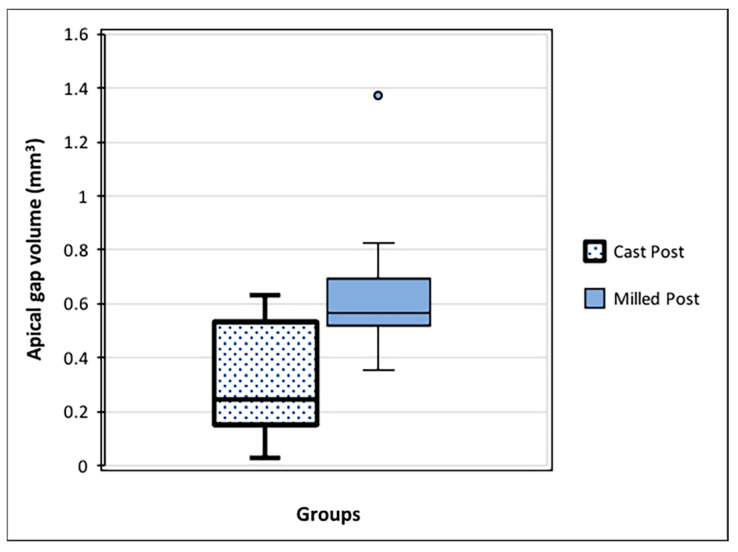
Apical gap volume (mm^3^) between the apical end of each post and the remaining gutta-percha root canal filling material. The milled post group had a larger space volume than the cast post group (*p* < 0.02).

The apical gap volume of the milled post group (1.029 ± 0.336 mm^3^) was higher than that of the cast post group (mean 0.346 ± 0.158 mm^3^), as shown in Figure 6. The reliability of the repeated measurements of the apical gap volume measurements was 0.943 [95% CI 0.912; 0.965]; *p* = 0.0001.

### 3.3. Comparison of Apical Gap Distance between the Two Groups

**Figure 7:**
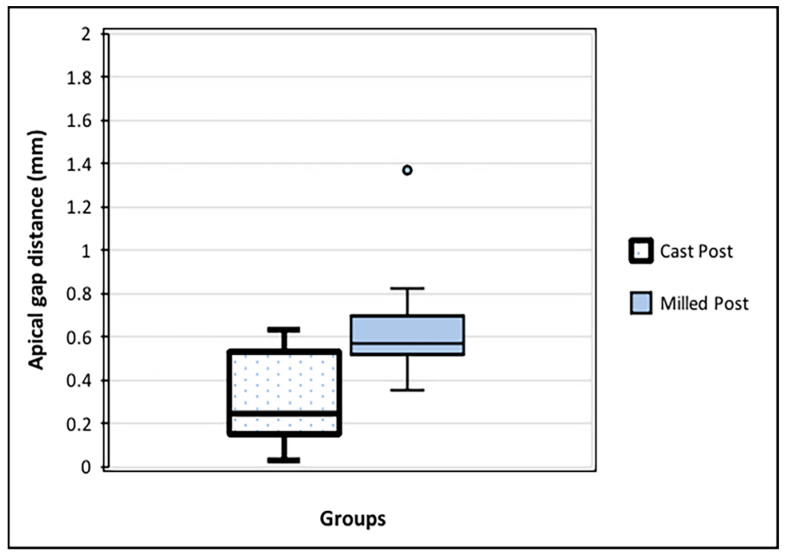
Apical gap distance (mm) between the apical end of each post and the remaining gutta-perch root canal filling material. The milled post group had a larger space volume than the cast post group (*p* < 0.02).

The apical gap distance of the milled post group (mean 0.622 ± 0.236 mm) was higher than that of the cast post group (mean 0.306 ± 0.204 mm), as shown in Figure 7. The reliability of the repeated measurements of the apical gap distance was 0.864 [95% CI 0.943; 0.821]; *p* = 0.001.

### 3.4. Comparison of Space between Posts and Lateral Walls of Root Canals

**Figure 8:**
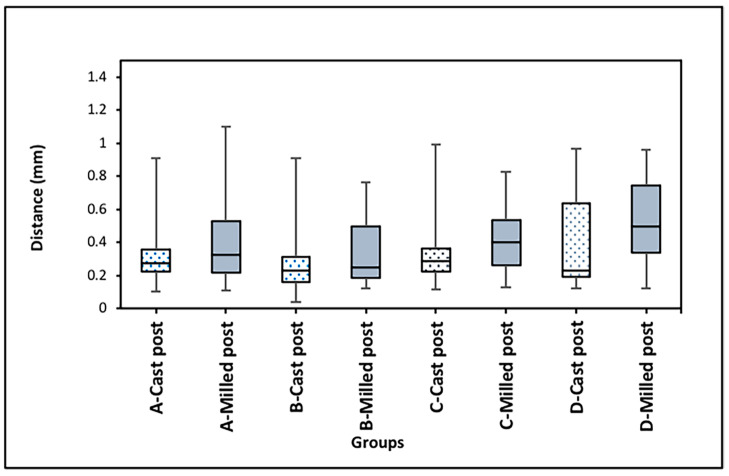
Distance between posts and lateral walls of root canal at four predetermined points (Point A, Point B, Point C, and Point D) along the length of each post (in mm). No significant differences between cast and milled posts were detected.

**Figure 9:**
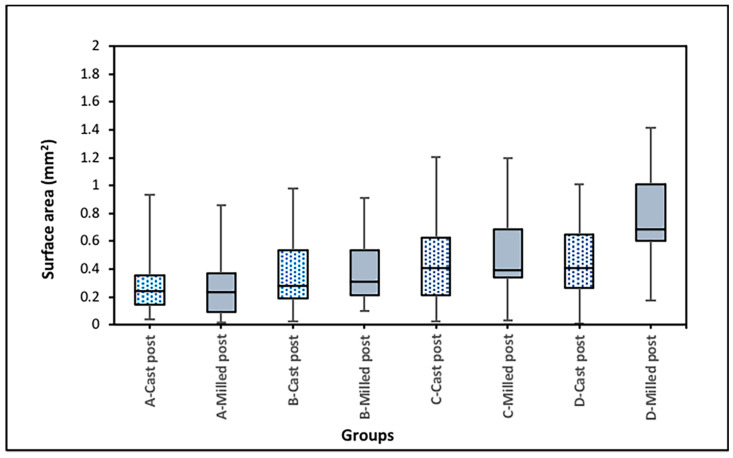
Surface areas between posts and lateral walls of root canal at four predetermined points (Point A, Point B, Point C, and Point D) along the length of each post (in mm^2^). Significant differences (*p* < 0.05) were revealed between milled and cast groups at point D, and between point D and points A and B within the milled group.

The means and standard deviations of the distances and surface areas between the posts and lateral surfaces of root canals as measured at four points along the length of posts are summarized in Table 1. Statistical analysis of the distance between the posts and root canal walls for the cast and milled posts at the predetermined four points along the length of each post did not reveal any significant differences (Table 1 and Figure 8). On the other hand, a significantly higher surface area was found in the milled post group as compared to the cast post group at point D (Table 1 and Figure 9). Furthermore, within groups, analysis demonstrated statistically significantly higher surface area values at D point as compared to A and B points within the milled post group (Table 1 and Figure 9). The reliability of the repeated measurement of distances along the lateral wall of posts was 0.815 [95% CI 0.906; 0.651]; *p* = 0.01. Meanwhile, that of the repeated measurements of surface areas was 0.876 [95% CI 0.931; 0.821]; *p* = 0.001.

## 4. Discussion

The null hypothesis was rejected, as the half-digital and milled post group was found to have significantly higher values for the total space volume, apical gap volume and distance, as well as a higher space surface area between the milled posts and prepared canal at the cervical area as compared to the cast post group.

The findings in this study that the half-digital and milled post group showed higher space values as compared to the cast post group could be the result of different reasons. The alloy used for the milled posts was reported to undergo a volumetric shrinkage of approximately 11%, as the organic binder burns out during sintering, forming metallic powder particles that are fused together without producing molten liquid [39]. In addition, it has been reported that half-digital milled posts and cores may have less accuracy due to sharp edges or small details that cannot be milled even with the smallest bur of the milling machine [9,40].

In comparison with other studies, the mean total space volume around cast posts observed in our study (mean 3.071 ± 1.56 mm^3^) was four times smaller than that reported (mean 12.25 ± 2.97 mm^3^) for cast posts made using direct technique by Rayyan and coworkers [17]. On the other hand, our results were slightly larger than those (mean 2.22 ± 1.35 mm^3^) reported by Kanduti and coworkers [38].

The different values of the total space volume reported in our study as compared to that of Kanduti and coworkers [38] could be the result of differences in the fabrication and fitting of cast posts in both studies. Before the application of a resin pattern to post space preparation, they [38] applied a thin layer of liquid paraffin, which could have produced a thinner layer than the petroleum jelly applied in our study as a separating medium. In addition, we have made every effort to reduce the thermal expansion of the post mold by controlling the liquid:powder ratio of the investment and burn out temperature and time, an issue that was not clearly described in their study. Furthermore, none of the cast posts in our study were miscast, and during the seating of posts, an adjustment of posts was performed until the core was seated, with finger pressure in the box made on the coronal part of each post. On the other hand, Kanduti and coworkers [38] indicated that each post was tested for a passive fit and that new posts were remade in case of discrepancies or failure, as judged by the investigators.

The mean total space volume of milled Co–Cr alloy posts made using half-digital technique in our study (mean 5.06 ± 1.917 mm^3^) was higher than that of Cr–Co alloy posts fabricated by full-digital and SLM technique (mean 3.82 ± 0.45 mm^3^) found in other studies [38]. The fabrication techniques of posts produced using a digital workflow in both studies were, however, different.

Studies have recommended that posts should be in contact with the remaining root canal filling and have suggested that as the gap between the tip of the post and the remaining root canal filling increases, the incidence of periapical disease increases [41]. In our study, it was found that the apical gap distance for cast posts was 0.306 ± 0.204 mm. These values were almost two times smaller than those (mean 0.61 ± 0.63 mm^3^) reported by Rayyan and coworkers [17] for cast posts when measurements were made using µCT.

In our study, the apical gap distance of half-digital and milled Cr–Co alloy posts was 0.622 ± 0.236 mm, which is similar to that (0.665 ± 0.189 mm) reported for cemented half-digital and milled posts when evaluated using digital radiographs [9]. The apical gap volume of half-digital milled posts, in our study, was 1.029 ± 0.3355 mm^3^; however, the apical gap volume for this type of post has not been reported in the literature.

The adaptation of posts to the lateral walls of post space preparation is believed to have a significant effect on the thickness of the luting cement [8], which in turn may have a detrimental effect on the retention and success of post and core restoration [6,7,9]. The distances and surface areas of the space between the posts and root canal, in our study, were measured on four points along the length of the post. The position of these points was similar for all posts, as µCT scans were performed using a positioning chamber and the area scanned was selected in the scout view to start at the most coronal part of the tooth root and extend to include the coronal end of the remaining gutta-perch root canal filling. The position of the four points at which measurements were made was standardized to start at the µCT slice that was 2.0 mm coronal to the most coronal end of the remaining gutta-percha root canal filling material. The remaining points were 1.5 mm apart. On the other hand, the distance and surface area space measurements along the side of cast posts were evaluated at four points along the length of the post in one study [38], whereas another study [17] reported only surface area measurements at three points along the length of posts.

For cast posts, the distances at the four points along the length of the post reported by Kanduti and coworkers [38] were smaller (85.73 ± 63.63 µ apically, 34.33 ± 17.60 and 36.76 ± 34.05 µ in the middle of the post, and 60.72 ± 32.51µ cervically) than the values found in our study (0.3755 ± 0.2614 mm apically, 0.3221 ± 0.2638 mm and 0.3951 ± 0.2961 mm in the middle of the post, and 0.4372 ± 0.1656 cervically). Furthermore, the surface areas of the space between the cast posts and surface of the root canal reported by the same investigators [38] were also smaller (0.25 ± 0.21 mm^2^ apically, 0.17 ± 0.07 mm^2^ and 0.12 ± 0.08 mm^2^ in the middle of the post, and 0.37 ± 0.21 mm^2^ cervically) than the surface areas along the side of the cast post found in our study (0.3218 ± 0.2658 mm^2^ apically, 0.3805 ± 0.2879 and 0.4759 ± 0.3616 mm^2^ in the middle of the post, and 0.4701 ± 0.3049 mm^2^ cervically). These differences could be the result of differences in the fabrication and fitting of the cast posts between both studies, as has been alluded to earlier.

Rayyan and coworkers [17] reported surface area measurements of the space between the cast posts and walls of root canals at three points along the length of the post (0.88 ± 0.53 mm^2^ apically, 1.20 ± 0.48 mm^2^ in the middle length of the post, and 1.18 ± 0.63 mm^2^ cervically) that were at least 2.5 times higher than the values found in our study. In their study [17], however, longer posts were used as compared to the posts used in our study. Furthermore, the position of the measurement points were determined using different criteria. These variations may account for the differences in the values of the surface areas reported in Rayyan and coworkers’ study [17] as compared to our study.

Retention of posts can be influenced by several factors, including post-related factors, tooth-related factors, and cementation-related factors. Regarding cementation-related factors, the type of cement, whether posts are luted or adhesively bonded, and the cement film thickness are variables that may affect the retention of posts [42,43]. For luting cements, the literature indicates that increasing the gap between the post and tooth structure results in an increased cement film thickness and decreased retention [43]. Using zinc phosphate luting cement, it was found that the highest retention values were obtained when the cement film thickness was about 65 µ as compared to those with 124 µ or 259 µ cement film thicknesses [43]. Hendi and coworkers [9] compared the retention of cast and half-digital milled Co–Cr alloy custom posts luted with zinc phosphate cement and found the cast posts to have a higher retention than the half-digital milled posts [9]. In that study [9], however, the gap between post and root canal was not evaluated. The gaps between posts and root canal walls found in our study were larger than the cement film thickness values recommended in the literature [43] for zinc phosphate cement.

On the other hand, the retention of adhesively bonded posts seems to be less affected by an increased resin cement film thickness [7,43,44,45] of up to 316.7 (± 58) µ [45]. These values for resin cement film thickness reported by Perez and coworkers [45] are similar to the values of space distance seen between the posts and walls of the root canal in the cast post group in our study. On the contrary, in our study, the values of space between the posts and root canal walls in the half-digital milled post group, particularly those at the cervical third of the milled post, appear to be higher than the recommended resin cement film thickness for optimum retention.

The success of any restoration is best confirmed in long-term clinical studies. However, our study was conducted in a well-controlled laboratory setting and aimed to evaluate the accuracy of fit of custom-made cast and half-digital and milled Co–Cr posts. Central incisors were selected because they have long roots in which root canals are round or oval in cross-section with fewer root canal variations. This helped in reducing variations in the size and shape of the post space preparations of different teeth. During the study, each tooth was used to evaluate both types of posts, which allowed control of confounding factors such as variations in the size and/or shape of posts. The use of one tooth to test the accuracy of fit of both posts used in this study precluded testing the retention of posts. The effect of fit on the retention of milled cast posts is being currently tested in another study. In addition, each tooth in this study was placed in a positioning chamber that allowed the standardization of the position of samples during µCT scanning and measurements. Furthermore, the use of µCT analysis allowed a nondestructive, high-resolution method for the assessment of the fit and accuracy of the investigated posts.

## 5. Conclusions

Based on the results of this study, it can be concluded that custom-made posts fabricated with half-digital and milled technique showed a higher total space volume, apical gap volume, apical gap distance and a larger space surface area between the milled post and prepared canal at the cervical area as compared to the cast post.

The clinical implications of these results indicate that custom-made Co–Cr posts fabricated using half-digital and milling technique have a less accurate internal fit as compared to custom-made cast posts fabricated using direct technique. Clinicians should take into consideration the accuracy of fit of custom-made posts during the selection of materials and techniques for the fabrication of posts.

## Figures and Tables

**Table 1:** Means and standard deviations of distance and surface area values between posts and lateral walls of root canals as measured at four predetermined points along the length of each post.

Measurement Point	Technique	Distance (mm)	Surface Area (mm^2^)
**Point A**	Milled post	0.395 ± 0.268	0.284 ± 0.245 ^b^
	Cast Post	0.376 ± 0.261	0.322 ± 0.266
**Point B**	Milled post	0.341 ± 0.202	0.423 ± 0.293 ^c^
	Cast Post	0.322 ± 0.264	0.381 ± 0.288
**Point C**	Milled post	0.421 ± 0.192	0.491 ± 0.310
	Cast Post	0.395 ± 0.296	0.476 ± 0.362
**Point D**	Milled post	0.532 ± 0.281	0.792 ± 0.378 ^a,b,c^
	Cast Post	0.399 ± 0.287	0.470 ± 0.305 ^a^

Similar letters are significantly different from each other. ^a^ *p* < 0.05; ^b^ *p* < 0.05; ^c^ *p* < 0.05.

## Data Availability

All data supporting the reported results are available upon request.
